# Crystal structure of 26-(4-methyl­phen­yl)-8,11,14,17-tetra­oxa-28-aza­tetra­cyclo[22.3.1.0^2,7^.0^18,23^]hexa­cosa-2,4,6,18(23),19,21,24(1),25,27-nona­ene

**DOI:** 10.1107/S2056989016005752

**Published:** 2016-04-12

**Authors:** T. Thanh Van Tran, Le Tuan Anh, Hung Huy Nguyen, Hong Hieu Truong, Anatoly T. Soldatenkov

**Affiliations:** a Faculty of Chemistry, University of Science, Vietnam National University, 19 Le Thanh Tong, Hanoi, Vietnam; b Institute of Chemistry, Vietnam Academy of Science and Technology, 18 Hoang Quoc Viet, Hanoi, Vietnam; c Organic Chemistry Department, Peoples Friendship University of Russia, Miklukho-Maklaya St. 6, Moscow 117198, Russian Federation

**Keywords:** crystal structure, 4-aryl­pyridine, aza-17-crown-5 ether, Chichibabin domino reaction, C—H⋯N hydrogen bonding, C—H⋯π inter­actions

## Abstract

The title compound, C_30_H_29_NO_4_, is a tetra­cyclic system containing a 4-aryl­pyridine fragment, two benzene rings and an aza-17-crown-5 ether moiety, in a bowl-like arrangement. The pyridine ring is inclined to the 4-methyl­phenyl ring by 26.64 (6)°, and by 57.43 (6) and 56.81 (6)° to the benzene rings. The benzene rings are inclined to one another by 88.32 (6)°. In the crystal, mol­ecules are linked by pairs of C—H⋯N hydrogen bonds, forming inversion dimers with an *R*_2_^2^(14) ring motif. The dimers are linked *via* a number of C—H⋯π inter­actions, forming a three-dimensional architecture.

## Abstract 2

The title compound is the product of the Chichibabin domino reaction of 1,8-bis­(2-acetyl­phen­oxy)-3,6-dioxa­octane with 4-methyl­benzaldehyde and ammonium acetate in acetic acid. It is of inter­est with respect to its potential anti­cancer activity. The compound has a bowl-like conformation comprising a fused tetra­cyclic system containing a 4-aryl­pyridine fragment, two benzene rings and an aza-17-crown-5 ether moiety.

## Chemical context   

Over the last decades, there has been considerable inter­est in pyridino-fused aza­crown ethers owing to their great theoret­ical and practical potential (Bradshaw *et al.*, 1993 ▸). Among them, pyridino­crownophanes containing a benzo subunit show high effectiveness as complexating ligands in metal-ion capture and separation (Pedersen, 1988 ▸). They are also of inter­est as phase-transfer catalysts, as membrane ion transporting vehicles (Gokel & Murillo, 1996 ▸), as active components useful in environmental chemistry (Bradshaw & Izatt, 1997 ▸), in design technology for the construction of organic sensors (Costero *et al.*, 2005 ▸) and as nanosized on–off switches and other mol­ecular electronic devices (Natali & Giordani, 2012 ▸). It has also been shown that the family of pyridino­aza­crown compounds can possess anti­bacterial (An *et al.*, 1998 ▸) and anti­cancer properties (Artiemenko *et al.*, 2002 ▸; Le *et al.*, 2015 ▸).

**Figure 1:**
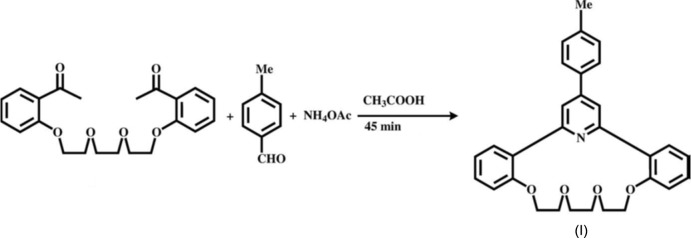
Chichibabin-type condensation of 1,8-bis­(2-acetyl­phen­oxy)-3,6-dioxa­octane with 4-methyl­benzaldehyde and ammonium acetate to produce the title compound (I).

Recently, we have proposed a new efficient one-step Chichibabin method for the preparation of a series of pyridino­crownophanes incorporating a 14-crown-4 ether moiety (Le *et al.*, 2014 ▸, 2015 ▸; Anh *et al.*, 2008 ▸; Levov *et al.*, 2008 ▸). During the course of our attempts to develop the chemistry of these aza­crown systems and obtain macrocyclic ligands which include more extended macro-heterocycles, namely the 17-crown-5 ether moiety, we have studied the Chichibabin-type condensation of 1,8-bis­(2-acetyl­phen­oxy)-3,6-dioxa­octane with 4-methyl­benzaldehyde and ammonium acetate in acetic acid. This reaction (Fig. 1 ▸) proceeds smoothly under heating of the multicomponent mixture to give the expected aza­crown with reasonable yield (30%). Herein, we report on the synthesis and crystal structure of this new aza­crown compound (I).
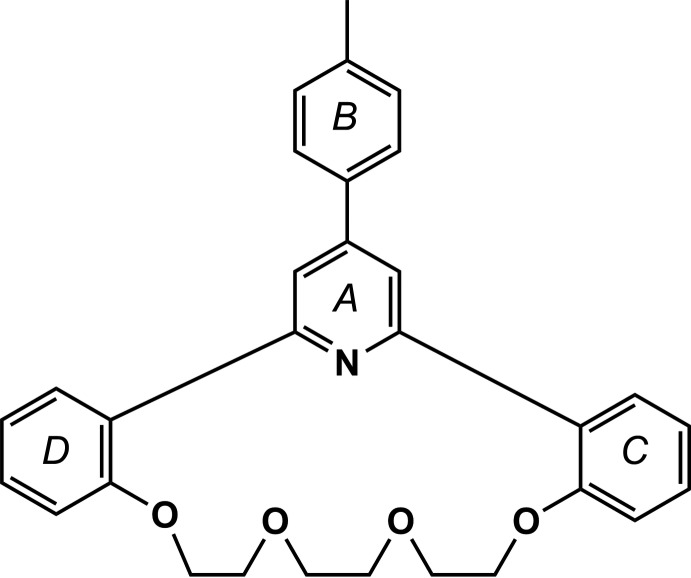


## Structural commentary   

**Figure 2:**
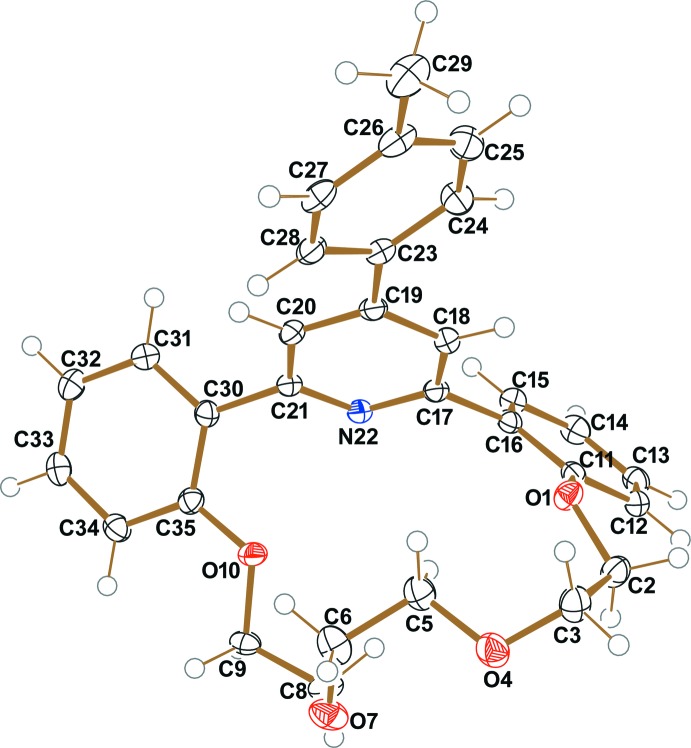
Mol­ecular structure of the title compound (I), with the atom labelling. Displacement ellipsoids are drawn at the 50% probability level.

The mol­ecule of the title compound, (I), is a tetra­cyclic system containing a 4-aryl­pyridine fragment (rings *A* = N22/C17–C22 and *B* = C23–C28), two benzene rings (*C* = C11–C16 and *D* = C30–C35), and an aza-17-crown-5 ether moiety, and has a bowl-like arrangement (Fig. 2 ▸). While the dihedral angles between the benzene rings and the pyridine ring are *A*/*D* = 56.81 (6)° and *A*/*C* = 57.43 (6)°, the dihedral angle between the 4-methyl­phenyl ring (*B*) and the pyridine ring (*A*) in the 4-aryl­pyridine fragment is only 26.64 (6)°. The distances from the center of the macrocycle cavity, defined as the centroid of atoms O1/O4/O7/O10/N22, to the individual atoms O1, O4, O7, O10 and N22 are 2.813 (2), 2.549 (2), 2.588 (2), 2.517 (2) and 2.825 (2) Å, respectively.

## Supra­molecular features   

**Figure 3:**
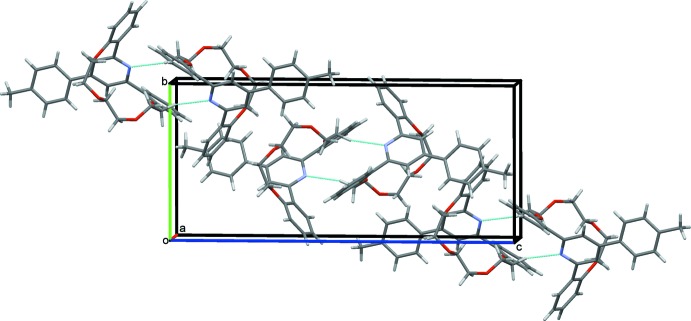
A view along the *a* axis of the crystal packing of the title compound (I). The C—H⋯N hydrogen bonds are shown as dashed lines (see Table 1 ▸).

In the crystal, mol­ecules are linked by pairs of C—H⋯N hydrogen bonds, forming inversion dimers with an 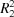
(14) ring motif (Table 1 ▸ and Fig. 3 ▸). The dimers are linked *via* a number of C—H⋯π inter­actions, forming a three-dimensional structure (Table 1 ▸).

## Database survey   

**Figure 4:**
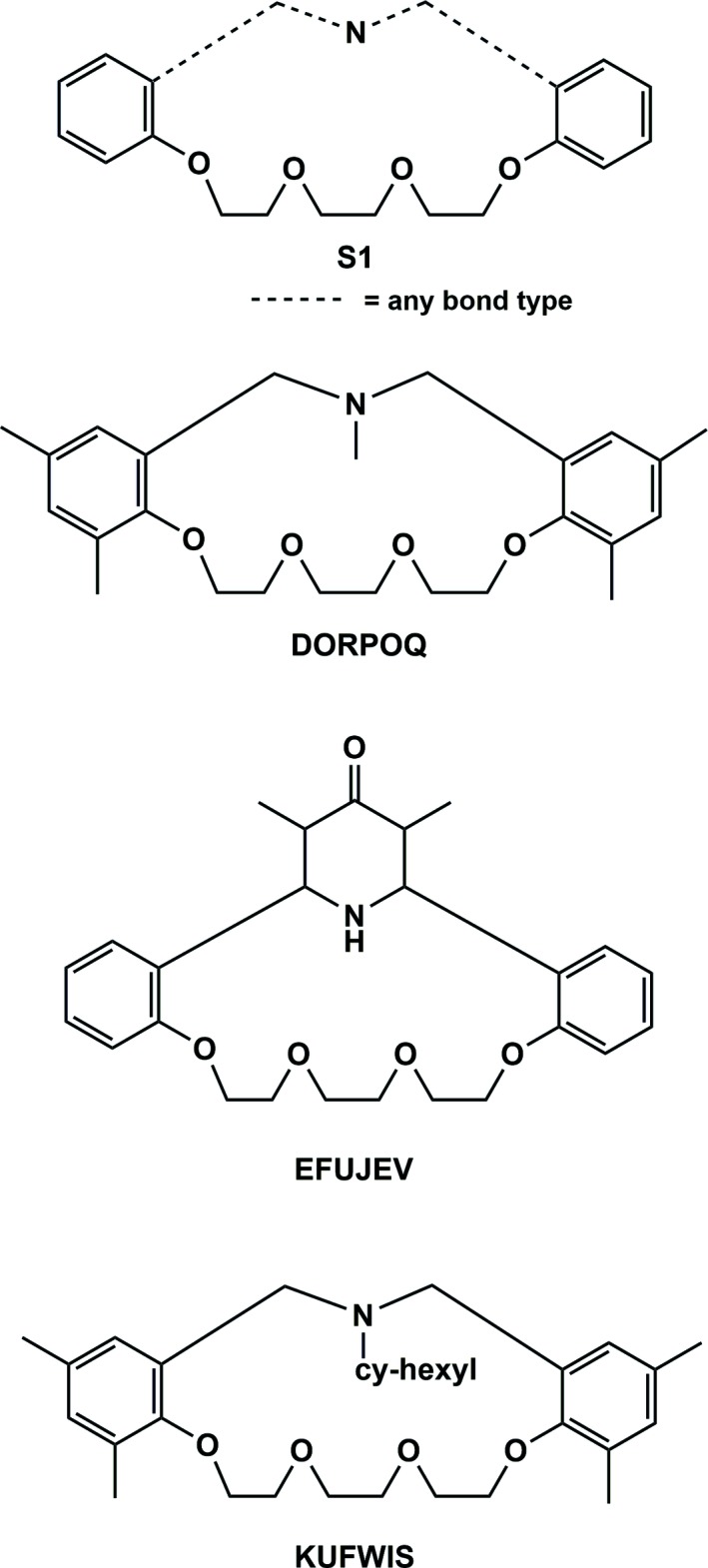
Database search substructure **S1**, and results.

A search of the Cambridge Structural Database (CSD, Version 5.38, update February 2016; Groom *et al.*, 2016 ▸) for the macrocyclic substructure **S1**, illustrated in Fig. 4 ▸, gave three hits, *viz*. 2,4,15,17,20-penta­methyl-6,7,9,10,12,13,20,21-octa­hydro-19*H-*dibenzo[*k*,*p*][1,4,7,10,14]tetra­oxaza­cyclohepta­decine (DORPOQ; Rungsimanon *et al.*, 2008 ▸), 25,27-dimethyl-8,11,14,17-tetra­oxa-28-aza­tetra­cyclo­(22.3.1.0^2,7^.0^18,23^)octa­cosa-2,4,6,18 (23),19,21-hexen-26-one (EFIJEV; Levov *et al.*, 2008 ▸), and 20-cyclo­hexyl-2,4,15,17-tetra­methyl-6,7,9,10,12,13,20,21-octa­hydro-19*H*-dibenzo[*k*,*p*][1,4,7,10,14]tetra­oxaza­cyclo­hepta­decine (KUFWIS; Chirachanchai *et al.*, 2009 ▸), also illustrated in Fig. 4 ▸. The two benzene rings are inclined to one another by 50.41 (6)° in DORPOQ, 88.28 (9)° in EFIJEV and 74.3 (9)° in KUGWIS. The corresponding dihedral angle in the title compound [*D*/*C* = 88.32 (6)°] is similar to that observed in EFIJEV.

## Synthesis and crystallization   

The synthesis of the title compound (I), is illustrated in Fig. 1 ▸. Ammonium acetate (10.0 g, 130 mmol) was added to a solution of 1,8-bis­(2-acetyl­phen­oxy)-3,6-dioxa­octane (0.50 g, 1.30 mmol) and *p*-methyl­benzaldehyde (0.155 g, 1.30 mmol) in acetic acid (10 ml). The reaction mixture was then refluxed for 45 min (monitored by TLC until disappearance of the starting diketone spot). At the end of the reaction, the reaction mixture was left to cool to room temperature, neutralized with Na_2_CO_3_ and extracted with ethyl acetate. The extract was purified by column chromatography on silica gel to give colourless crystals of the title compound (I) [yield 0.18 g, 30%; m.p. 471–472 K]. IR (KBr), ν cm^−1^: C=N_pyridine_ (1607), C=C_aromatic_ (1545, 1514, 1492), C—O—C (1182, 1120, 1058, 1029). ^1^H NMR (CDCl_3_, 500 MHz, 300 K): *d* = 2.42 (*s*, 3H, CH_3_), 3.18 (*s*, 4H, H_ether_), 3.62 and 4.11 (both *t*, 4H each, H_ether_, *J* = 8 Hz each), 7.0–6.98 (*d*, 2H, H_arom_), 7.13–7.10 (*m*, 2H, H_arom_), 7.30–7.29 (*d*, 2H, H_arom_), 7.37–7.34 (*m*, 2H, H_arom_), 7.66–7.62 (*m*, 4H, H_arom_), 7.75 (*s*, 2H, H_25, 27_). ESI–MS: [*M* + H]^+^ = 468.2. Analysis calculated for C_30_H_29_NO_4_: C, 77.07; H, 6.25; N, 3.00. Found: C, 77.22; H, 6.05; N, 3.12.

## Refinement   

Crystal data, data collection and structure refinement details are summarized in Table 2 ▸. The H atoms were placed in calculated positions and refined as riding atoms: C—H = 0.95–0.99 Å with *U*_iso_(H) = 1.5*U*_eq_(C-meth­yl) and 1.2*U*_eq_(C) for other H atoms.

## Supplementary Material

Crystal structure: contains datablock(s) Global, I. DOI: 10.1107/S2056989016005752/su5294sup1.cif

Structure factors: contains datablock(s) I. DOI: 10.1107/S2056989016005752/su5294Isup2.hkl

CCDC reference: 1472697

Additional supporting information:  crystallographic information; 3D view; checkCIF report

## Figures and Tables

**Table 1:** Hydrogen-bond geometry (Å, °) *Cg*1, *Cg*2, *Cg*3 and *Cg*4 are the centroids of rings *A* (N22/C17–C21), *C* (C11–C16), *B* (C23–C28) and *D* (C30–C35), respectively.

*D*—H⋯*A*	*D*—H	H⋯*A*	*D*⋯*A*	*D*—H⋯*A*
C9—H9*A*⋯N22^i^	0.99	2.55	3.4606 (15)	152
C3—H3*B*⋯*Cg*2^ii^	0.99	2.75	3.6182 (15)	146
C12—H12⋯*Cg*3^iii^	0.95	2.93	3.7281 (13)	142
C25—H25⋯*Cg*4^iv^	0.95	2.86	3.6987 (15)	148
C27—H27⋯*Cg*1^v^	0.95	2.99	3.7685 (14)	140
C34—H34⋯*Cg*2^i^	0.95	2.77	3.5912 (13)	146

Symmetry codes: (i) 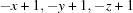; (ii) 
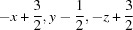
; (iii) 
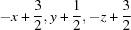
; (iv) 
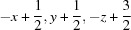
; (v) 
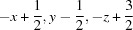
.

**Table 2:** Experimental details

Crystal data	
Chemical formula	C_30_H_29_NO_4_
*M*_r_	467.54
Crystal system, space group	Monoclinic, *P*2_1_/*n*
Temperature (K)	100
*a*, *b*, *c* (Å)	10.0819 (4), 10.4531 (4), 23.6016 (9)
β (°)	100.607 (1)
*V* (Å^3^)	2444.80 (16)
*Z*	4
Radiation type	Mo *K*α
μ (mm^−1^)	0.08
Crystal size (mm)	0.14 × 0.12 × 0.12

Data collection	
Diffractometer	D8 Quest Bruker CMOS
Absorption correction	Multi-scan (*SADABS*; Bruker, 2014 ▸)
*T*_min_, *T*_max_	0.695, 0.746
No. of measured, independent and observed [*I* > 2σ(*I*)] reflections	77012, 5825, 4706
*R*_int_	0.043
(sin θ/λ)_max_ (Å^−1^)	0.658

Refinement	
*R*[*F* ^2^ > 2σ(*F* ^2^)], *wR*(*F* ^2^), *S*	0.040, 0.099, 1.01
No. of reflections	5825
No. of parameters	317
H-atom treatment	H-atom parameters constrained
Δρ_max_, Δρ_min_ (e Å^−3^)	0.31, −0.20

Computer programs: *APEX2* and *SAINT* (Bruker, 2014 ▸), *SHELXT2014* (Sheldrick, 2015*a* ▸), *SHELXL2014* (Sheldrick, 2015*b* ▸), *OLEX2* (Dolomanov *et al.*, 2009 ▸), *Mercury* (Macrae *et al.*, 2008 ▸) and *PLATON* (Spek, 2009 ▸).

## supplementary crystallographic information

### Crystal data

**Table:**

C_30_H_29_NO_4_	*F*(000) = 992
*M**_r_* = 467.54	*D*_x_ = 1.270 Mg m^−^^3^
Monoclinic, *P*2_1_/*n*	Mo *K*α radiation, λ = 0.71073 Å
*a* = 10.0819 (4) Å	Cell parameters from 9281 reflections
*b* = 10.4531 (4) Å	θ = 2.9–28.3°
*c* = 23.6016 (9) Å	µ = 0.08 mm^−^^1^
β = 100.607 (1)°	*T* = 100 K
*V* = 2444.80 (16) Å^3^	Block, colourless
*Z* = 4	0.14 × 0.12 × 0.12 mm

### Data collection

**Table:**

D8 Quest Bruker CMOS diffractometer	4706 reflections with *I* > 2σ(*I*)
Detector resolution: 0.5 pixels mm^-1^	*R*_int_ = 0.043
ω and φ scans	θ_max_ = 27.9°, θ_min_ = 2.8°
Absorption correction: multi-scan (*SADABS*; Bruker, 2014)	*h* = −13→13
*T*_min_ = 0.695, *T*_max_ = 0.746	*k* = −13→13
77012 measured reflections	*l* = −31→30
5825 independent reflections	

### Refinement

**Table:**

Refinement on *F*^2^	0 restraints
Least-squares matrix: full	Hydrogen site location: inferred from neighbouring sites
*R*[*F*^2^ > 2σ(*F*^2^)] = 0.040	H-atom parameters constrained
*wR*(*F*^2^) = 0.099	*w* = 1/[σ^2^(*F*_o_^2^) + (0.0422*P*)^2^ + 1.1744*P*] where *P* = (*F*_o_^2^ + 2*F*_c_^2^)/3
*S* = 1.01	(Δ/σ)_max_ = 0.001
5825 reflections	Δρ_max_ = 0.31 e Å^−^^3^
317 parameters	Δρ_min_ = −0.19 e Å^−^^3^

### Special details

**Table:**

Geometry. All esds (except the esd in the dihedral angle between two l.s. planes) are estimated using the full covariance matrix. The cell esds are taken into account individually in the estimation of esds in distances, angles and torsion angles; correlations between esds in cell parameters are only used when they are defined by crystal symmetry. An approximate (isotropic) treatment of cell esds is used for estimating esds involving l.s. planes.

### Fractional atomic coordinates and isotropic or equivalent isotropic displacement parameters (Å^2^)

**Table:**

	*x*	*y*	*z*	*U*_iso_*/*U*_eq_	
O1	0.76598 (8)	0.67651 (8)	0.70887 (4)	0.02073 (19)	
C2	0.90811 (12)	0.64977 (12)	0.71765 (6)	0.0236 (3)	
H2A	0.9585	0.7123	0.7450	0.028*	
H2B	0.9407	0.6561	0.6807	0.028*	
C3	0.93002 (13)	0.51576 (13)	0.74174 (6)	0.0260 (3)	
H3A	1.0261	0.5060	0.7600	0.031*	
H3B	0.8752	0.5042	0.7721	0.031*	
O4	0.89565 (10)	0.41796 (9)	0.69952 (4)	0.0283 (2)	
C5	0.75342 (13)	0.40022 (13)	0.68115 (6)	0.0254 (3)	
H5A	0.7148	0.4735	0.6570	0.030*	
H5B	0.7088	0.3956	0.7151	0.030*	
C6	0.72925 (15)	0.27780 (13)	0.64690 (6)	0.0276 (3)	
H6A	0.7722	0.2065	0.6712	0.033*	
H6B	0.6310	0.2610	0.6382	0.033*	
O7	0.77874 (10)	0.27738 (9)	0.59417 (4)	0.0294 (2)	
C8	0.71355 (13)	0.36342 (13)	0.55114 (5)	0.0240 (3)	
H8A	0.7674	0.3683	0.5201	0.029*	
H8B	0.7137	0.4497	0.5685	0.029*	
C9	0.57024 (12)	0.32947 (12)	0.52436 (5)	0.0191 (2)	
H9A	0.5462	0.3679	0.4855	0.023*	
H9B	0.5602	0.2355	0.5206	0.023*	
O10	0.48389 (8)	0.37838 (9)	0.56118 (4)	0.0226 (2)	
C11	0.71707 (12)	0.77257 (11)	0.67121 (5)	0.0169 (2)	
C12	0.79826 (12)	0.86832 (12)	0.65433 (5)	0.0202 (2)	
H12	0.8924	0.8694	0.6694	0.024*	
C13	0.74163 (13)	0.96191 (12)	0.61564 (5)	0.0216 (3)	
H13	0.7973	1.0267	0.6041	0.026*	
C14	0.60453 (13)	0.96142 (12)	0.59371 (5)	0.0223 (3)	
H14	0.5656	1.0261	0.5675	0.027*	
C15	0.52418 (12)	0.86528 (12)	0.61036 (5)	0.0200 (2)	
H15	0.4303	0.8646	0.5949	0.024*	
C16	0.57788 (12)	0.77018 (11)	0.64904 (5)	0.0167 (2)	
C17	0.48787 (11)	0.66625 (11)	0.66367 (5)	0.0164 (2)	
C18	0.47440 (12)	0.64112 (11)	0.72038 (5)	0.0177 (2)	
H18	0.5257	0.6882	0.7513	0.021*	
C19	0.38500 (11)	0.54616 (11)	0.73144 (5)	0.0173 (2)	
C20	0.31490 (12)	0.47876 (11)	0.68404 (5)	0.0182 (2)	
H20	0.2526	0.4137	0.6895	0.022*	
C21	0.33672 (11)	0.50726 (11)	0.62894 (5)	0.0170 (2)	
N22	0.41964 (10)	0.60176 (9)	0.61821 (4)	0.0168 (2)	
C23	0.36716 (11)	0.51592 (12)	0.79121 (5)	0.0182 (2)	
C24	0.39161 (13)	0.60818 (13)	0.83481 (5)	0.0233 (3)	
H24	0.4182	0.6920	0.8261	0.028*	
C25	0.37740 (14)	0.57841 (14)	0.89078 (5)	0.0264 (3)	
H25	0.3930	0.6428	0.9196	0.032*	
C26	0.34073 (12)	0.45582 (14)	0.90541 (5)	0.0243 (3)	
C27	0.31736 (12)	0.36405 (13)	0.86220 (6)	0.0231 (3)	
H27	0.2930	0.2797	0.8713	0.028*	
C28	0.32895 (12)	0.39334 (12)	0.80585 (5)	0.0207 (3)	
H28	0.3107	0.3293	0.7769	0.025*	
C29	0.32647 (14)	0.42573 (16)	0.96669 (6)	0.0331 (3)	
H29A	0.4159	0.4246	0.9914	0.050*	
H29B	0.2837	0.3418	0.9679	0.050*	
H29C	0.2705	0.4913	0.9804	0.050*	
C30	0.26916 (12)	0.43182 (11)	0.57809 (5)	0.0177 (2)	
C31	0.12989 (13)	0.42073 (12)	0.56426 (6)	0.0241 (3)	
H31	0.0755	0.4597	0.5883	0.029*	
C32	0.06873 (13)	0.35301 (13)	0.51548 (6)	0.0279 (3)	
H32	−0.0268	0.3469	0.5062	0.034*	
C33	0.14684 (13)	0.29501 (12)	0.48079 (6)	0.0240 (3)	
H33	0.1047	0.2502	0.4473	0.029*	
C34	0.28687 (13)	0.30147 (12)	0.49435 (5)	0.0208 (3)	
H34	0.3404	0.2600	0.4707	0.025*	
C35	0.34786 (12)	0.36931 (11)	0.54301 (5)	0.0180 (2)	

### Atomic displacement parameters (Å^2^)

**Table:**

	*U*^11^	*U*^22^	*U*^33^	*U*^12^	*U*^13^	*U*^23^
O1	0.0174 (4)	0.0189 (4)	0.0258 (4)	−0.0004 (3)	0.0037 (3)	0.0035 (3)
C2	0.0177 (6)	0.0225 (6)	0.0299 (7)	−0.0001 (5)	0.0023 (5)	0.0013 (5)
C3	0.0239 (6)	0.0238 (7)	0.0276 (7)	0.0010 (5)	−0.0024 (5)	0.0017 (5)
O4	0.0277 (5)	0.0221 (5)	0.0332 (5)	0.0041 (4)	0.0006 (4)	−0.0031 (4)
C5	0.0277 (7)	0.0229 (6)	0.0242 (6)	0.0009 (5)	0.0012 (5)	−0.0007 (5)
C6	0.0370 (8)	0.0201 (6)	0.0235 (6)	0.0021 (6)	−0.0002 (5)	0.0027 (5)
O7	0.0303 (5)	0.0283 (5)	0.0280 (5)	0.0122 (4)	0.0016 (4)	−0.0012 (4)
C8	0.0213 (6)	0.0281 (7)	0.0244 (6)	0.0036 (5)	0.0092 (5)	0.0040 (5)
C9	0.0227 (6)	0.0193 (6)	0.0174 (5)	0.0030 (5)	0.0090 (5)	0.0013 (5)
O10	0.0162 (4)	0.0311 (5)	0.0214 (4)	0.0001 (4)	0.0057 (3)	−0.0089 (4)
C11	0.0196 (6)	0.0140 (5)	0.0176 (5)	−0.0007 (4)	0.0050 (4)	−0.0024 (4)
C12	0.0189 (6)	0.0180 (6)	0.0247 (6)	−0.0031 (5)	0.0067 (5)	−0.0046 (5)
C13	0.0281 (6)	0.0150 (6)	0.0245 (6)	−0.0049 (5)	0.0120 (5)	−0.0021 (5)
C14	0.0288 (7)	0.0174 (6)	0.0216 (6)	0.0010 (5)	0.0071 (5)	0.0030 (5)
C15	0.0198 (6)	0.0202 (6)	0.0205 (6)	−0.0005 (5)	0.0046 (5)	−0.0015 (5)
C16	0.0195 (6)	0.0156 (5)	0.0167 (5)	−0.0021 (4)	0.0075 (4)	−0.0029 (4)
C17	0.0148 (5)	0.0156 (5)	0.0195 (6)	0.0008 (4)	0.0052 (4)	−0.0008 (4)
C18	0.0173 (5)	0.0180 (6)	0.0179 (5)	−0.0012 (4)	0.0040 (4)	−0.0019 (4)
C19	0.0153 (5)	0.0181 (6)	0.0195 (6)	0.0017 (4)	0.0060 (4)	−0.0002 (4)
C20	0.0163 (5)	0.0172 (6)	0.0229 (6)	−0.0023 (4)	0.0078 (4)	−0.0012 (5)
C21	0.0146 (5)	0.0165 (6)	0.0207 (6)	0.0006 (4)	0.0055 (4)	−0.0019 (4)
N22	0.0159 (5)	0.0166 (5)	0.0188 (5)	−0.0002 (4)	0.0055 (4)	−0.0014 (4)
C23	0.0141 (5)	0.0219 (6)	0.0191 (6)	0.0009 (4)	0.0047 (4)	0.0020 (5)
C24	0.0280 (7)	0.0218 (6)	0.0208 (6)	−0.0014 (5)	0.0064 (5)	0.0016 (5)
C25	0.0297 (7)	0.0312 (7)	0.0186 (6)	−0.0020 (6)	0.0050 (5)	−0.0008 (5)
C26	0.0160 (6)	0.0366 (7)	0.0204 (6)	0.0007 (5)	0.0037 (5)	0.0076 (5)
C27	0.0167 (6)	0.0256 (6)	0.0282 (7)	−0.0015 (5)	0.0069 (5)	0.0082 (5)
C28	0.0158 (6)	0.0223 (6)	0.0252 (6)	−0.0015 (5)	0.0063 (5)	0.0006 (5)
C29	0.0280 (7)	0.0489 (9)	0.0219 (7)	−0.0044 (6)	0.0034 (5)	0.0117 (6)
C30	0.0188 (6)	0.0154 (5)	0.0193 (6)	−0.0021 (4)	0.0047 (4)	−0.0008 (4)
C31	0.0199 (6)	0.0240 (6)	0.0298 (7)	−0.0015 (5)	0.0083 (5)	−0.0059 (5)
C32	0.0171 (6)	0.0302 (7)	0.0351 (7)	−0.0035 (5)	0.0013 (5)	−0.0064 (6)
C33	0.0257 (6)	0.0225 (6)	0.0223 (6)	−0.0050 (5)	0.0004 (5)	−0.0042 (5)
C34	0.0247 (6)	0.0195 (6)	0.0190 (6)	−0.0009 (5)	0.0061 (5)	−0.0020 (5)
C35	0.0180 (6)	0.0183 (6)	0.0183 (6)	−0.0019 (4)	0.0049 (4)	0.0008 (4)

### Geometric parameters (Å, º)

**Table:**

O1—C2	1.4370 (15)	C18—C19	1.3973 (16)
O1—C11	1.3712 (14)	C19—C20	1.3987 (17)
C2—C3	1.5126 (18)	C19—C23	1.4886 (16)
C3—O4	1.4251 (16)	C20—C21	1.3905 (16)
O4—C5	1.4317 (16)	C21—N22	1.3479 (15)
C5—C6	1.5093 (18)	C21—C30	1.4917 (16)
C6—O7	1.4236 (17)	C23—C24	1.3987 (17)
O7—C8	1.4235 (15)	C23—C28	1.3995 (17)
C8—C9	1.5089 (17)	C24—C25	1.3900 (17)
C9—O10	1.4329 (14)	C25—C26	1.3946 (19)
O10—C35	1.3628 (14)	C26—C27	1.3883 (19)
C11—C12	1.3966 (16)	C26—C29	1.5125 (17)
C11—C16	1.4047 (16)	C27—C28	1.3905 (17)
C12—C13	1.3874 (18)	C30—C31	1.3867 (17)
C13—C14	1.3839 (18)	C30—C35	1.4084 (16)
C14—C15	1.3921 (17)	C31—C32	1.3947 (18)
C15—C16	1.3901 (17)	C32—C33	1.3768 (19)
C16—C17	1.4962 (16)	C33—C34	1.3906 (18)
C17—C18	1.3948 (16)	C34—C35	1.3928 (17)
C17—N22	1.3444 (15)		
			
C11—O1—C2	117.77 (9)	C20—C19—C23	121.31 (11)
O1—C2—C3	107.88 (10)	C21—C20—C19	119.77 (11)
O4—C3—C2	113.69 (11)	C20—C21—C30	120.79 (10)
C3—O4—C5	113.93 (10)	N22—C21—C20	122.89 (11)
O4—C5—C6	109.02 (11)	N22—C21—C30	116.32 (10)
O7—C6—C5	115.01 (11)	C17—N22—C21	117.48 (10)
C8—O7—C6	115.57 (10)	C24—C23—C19	121.01 (11)
O7—C8—C9	115.53 (11)	C24—C23—C28	117.96 (11)
O10—C9—C8	107.68 (10)	C28—C23—C19	121.01 (11)
C35—O10—C9	118.22 (9)	C25—C24—C23	120.65 (12)
O1—C11—C12	123.37 (11)	C24—C25—C26	121.29 (12)
O1—C11—C16	116.35 (10)	C25—C26—C29	120.26 (13)
C12—C11—C16	120.28 (11)	C27—C26—C25	118.03 (12)
C13—C12—C11	120.08 (11)	C27—C26—C29	121.71 (13)
C14—C13—C12	120.35 (11)	C26—C27—C28	121.18 (12)
C13—C14—C15	119.34 (11)	C27—C28—C23	120.87 (12)
C16—C15—C14	121.68 (11)	C31—C30—C21	121.75 (11)
C11—C16—C17	122.26 (10)	C31—C30—C35	118.58 (11)
C15—C16—C11	118.26 (11)	C35—C30—C21	119.67 (10)
C15—C16—C17	119.43 (11)	C30—C31—C32	120.76 (12)
C18—C17—C16	121.91 (10)	C33—C32—C31	120.00 (12)
N22—C17—C16	115.01 (10)	C32—C33—C34	120.62 (12)
N22—C17—C18	123.06 (11)	C33—C34—C35	119.34 (11)
C17—C18—C19	119.54 (11)	O10—C35—C30	115.21 (10)
C18—C19—C20	117.18 (11)	O10—C35—C34	124.11 (11)
C18—C19—C23	121.50 (11)	C34—C35—C30	120.66 (11)

### Hydrogen-bond geometry (Å, º)

Cg1, Cg2, Cg3 and Cg4 are the centroids of rings *A* (N22/C17–C21), *C* (C11–C16), *B* (C23–C28) and *D* (C30–C35), respectively.

**Table:**

*D*—H···*A*	*D*—H	H···*A*	*D*···*A*	*D*—H···*A*
C9—H9*A*···N22^i^	0.99	2.55	3.4606 (15)	152
C3—H3*B*···*Cg*2^ii^	0.99	2.75	3.6182 (15)	146
C12—H12···*Cg*3^iii^	0.95	2.93	3.7281 (13)	142
C25—H25···*Cg*4^iv^	0.95	2.86	3.6987 (15)	148
C27—H27···*Cg*1^v^	0.95	2.99	3.7685 (14)	140
C34—H34···*Cg*2^i^	0.95	2.77	3.5912 (13)	146

Symmetry codes: (i) −*x*+1, −*y*+1, −*z*+1; (ii) −*x*+3/2, *y*−1/2, −*z*+3/2; (iii) −*x*+3/2, *y*+1/2, −*z*+3/2; (iv) −*x*+1/2, *y*+1/2, −*z*+3/2; (v) −*x*+1/2, *y*−1/2, −*z*+3/2.
